# ﻿Five new species of Schizoporaceae (Basidiomycota, Hymenochaetales) from East Asia

**DOI:** 10.3897/mycokeys.96.99327

**Published:** 2023-03-14

**Authors:** Qian-Xin Guan, Jing Huang, Jian Huang, Chang-Lin Zhao

**Affiliations:** 1 College of Biodiversity Conservation, Southwest Forestry University, Kunming 650224, China; 2 Yunnan General Administration of Forestry Seeds and Seedlings, Kunming, 650215, China; 3 Yunnan Academy of Biodiversity, Southwest Forestry University, Kunming 650224, China; 4 Yunnan Key Laboratory for Fungal Diversity and Green Development, Kunming Institute of Botany, Chinese Academy of Science, Kunming 650201, China

**Keywords:** Biodiversity, China, molecular systematics, taxonomy, wood-inhabiting fungi, Yunnan Province

## Abstract

Five new wood-inhabiting fungi, *Lyomycesalbopulverulentus*, *L.yunnanensis*, *Xylodondaweishanensis*, *X.fissuratus*, and *X.puerensis***spp. nov.**, are proposed based on a combination of morphological features and molecular evidence. *Lyomycesalbopulverulentus* is characterized by brittle basidiomata, pruinose hymenophore with a white hymenial surface, a monomitic hyphal system with clamped generative hyphae, and ellipsoid basidiospores. *Lyomycesyunnanensis* is characterized by a grandinioid hymenial surface, the presence of capitate cystidia, and ellipsoid basidiospores. *Xylodondaweishanensis* is characterized by an odontioid hymenial surface, a monomitic hyphal system with clamped generative hyphae, and broad ellipsoid-to-subglobose basidiospores. *Xylodonfissuratus* is characterized by a cracking basidiomata with a grandinioid hymenial surface, and ellipsoid basidiospores. *Xylodonpuerensis* is characterized by a poroid hymenophore with an angular or slightly daedaleoid configuration, and ellipsoid-to-broad-ellipsoid basidiospores. Sequences of ITS and nLSU rRNA markers of the studied samples were generated and phylogenetic analyses were performed with the maximum likelihood, maximum parsimony, and Bayesian inference methods. The phylogram based on the ITS+nLSU rDNA gene regions (Fig. 1) included six genera within the families Chaetoporellaceae, Hyphodontiaceae, Hymenochaetaceae, and Schizoporaceae (Hymenochaetales)—*Fasciodontia*, *Hastodontia*, *Hyphodontia*, *Kneifiella*, *Lyomyces*, and *Xylodon*—in which the five new species were grouped into genera *Lyomyces* and *Xylodon*. The phylogenetic tree inferred from the ITS sequences highlighted that *Lyomycesalbopulverulentus* formed a monophyletic lineage and was then grouped closely with *L.bambusinus*, *L.orientalis*, and *L.sambuci*; additionally, *L.yunnanensis* was sister to *L.niveus* with strong supports. The topology, based on the ITS sequences, revealed that *Xylodondaweishanensis* was retrieved as a sister to *X.hyphodontinus*; *X.fissuratus* was grouped with the four taxa *X.montanus*, *X.subclavatus*, *X.wenshanensis*, and *X.xinpingensis*; and *X.puerensis* was clustered with *X.flaviporus*, *X.ovisporus*, *X.subflaviporus*, *X.subtropicus*, and *X.taiwanianus*.

## ﻿Introduction

Fungi represent one of the most diverse groups of organisms on the earth, with an indispensable role in the processes and functioning of ecosystems (Hyde 2022). The family Schizoporaceae Jülich includes many variations of the fruiting body types among the order Hymenochaetales Oberw. (Larsson et al. 2006; Wu et al. 2022a), in which it comprises many representative wood-inhabiting fungal taxa, including hydnoid, corticioid, and polyporoid basidiomes with diverse hymenophoral and cystidial morphology (Yurchenko and Wu 2016; Riebesehl and Langer 2017; Yurchenko et al. 2017; Cui et al. 2019; Riebesehl et al. 2019; Jiang et al. 2021; Wu et al. 2022a, b). In addition, the species of Schizoporaceae have been described from different countries, and they cause a white rot (Langer 1994).

The genus *Lyomyces* P. Karst. is a small corticioid group, typified by *L.sambuci* (Pers.) P. Karst. It is characterized by the resupinate-to-effused basidiomata with a smooth-to-odontioid hymenophore, a monomitic hyphal system with generative hyphae bearing clamp connections, the presence of several types of cystidia, and with smooth, thin- to slightly thick-walled basidiospores (Karsten 1881; Bernicchia and Gorjón 2010). The species of *Lyomyces* are found on fallen angiosperm branches, dead woody or herbaceous stems, or, occasionally, on gymnosperm wood (Yurchenko et al. 2017). Approximately 40 species of *Lyomyces* are currently known (Rabenhorst 1851; Karsten 1881, 1882; Cunningham 1959, 1963; Wu 1990; Hjortstam and Ryvarden 2009; Xiong et al. 2009; Dai 2010, 2011; Yurchenko et al. 2013, 2017, 2020; Gafforov et al. 2017; Riebesehl and Langer 2017; Chen and Zhao 2020; Luo et al. 2021b, c; Viner et al. 2022). The genus *Xylodon* (Pers.) Gray is typiﬁed by *X.quercinus* (Pers.) Gray (Bernicchia and Gorjón 2010). The taxa of this genus grow on rotten gymnosperm or angiosperm trunks and stumps, bamboo and ferns (Girometta et al. 2020; Greslebin and Rajchenberg 2000; Kotiranta and Saarenoksa 2000). They are characterized by resupinate or effuse basidiomata with a smooth, tuberculate, grandinioid, odontioid, coralloid, irpicoid, or poroid hymenophore; a monomitic or dimitic hyphal system with clamped generative hyphae; the presence of different types of cystidia, i.e., utriform or suburniform basidia; and cylindrical-to-ellipsoid-to-globose basidiospores, which can cause a white rot (Gray 1821; Bernicchia and Gorjón 2010). Based on the MycoBank database (http://www.mycobank.org, accessed on 24 December 2022) and the Index Fungorum (http://www.indexfungorum.org, accessed on 24 December 2022), *Xylodon* has registered 221 speciﬁc and infraspeciﬁc names, and the actual number of the species has reached 100 species (Chevallier 1826; Kuntze 1898; Wu 1990, 2000, 2001, 2006; Hjortstam and Ryvarden 2007, 2009; Xiong et al. 2009, 2010; Bernicchia and Gorjón 2010; Tura et al. 2011; Dai 2012; Lee and Langer 2012; Yurchenko et al. 2013; Yurchenko and Wu 2014; Zhao et al. 2014; Chen et al. 2016; Kan et al. 2017a, b; Riehl and Langer 2017; Wang and Chen 2017; Viner et al. 2018, 2022; Riebesehl et al. 2019; Shi et al. 2019; Dai et al. 2021; Luo et al. 2021a, 2022; Qu and Zhao 2022; Qu et al. 2022).

Classification of the kingdom of fungi has been updated continuously, based on the frequent inclusion of data from DNA sequences in many phylogenetic studies (Wijayawardene et al. 2020). Based on the early embrace of molecular systematics by mycologists, both the discovery and classiﬁcation of fungi, among the more basal branches of the tree, are now coming to light from genomic analyses and environmental DNA surveys that have been conducted (James et al. 2020). *Hyphodontia* s.l. was indicated to be a polyphyletic group, and *Xylodon* and *Kneiffiella* P. Karst. included the most species (Yurchenko and Wu 2016; Riebesehl and Langer 2017; Riebesehl et al. 2019). Given the lack of sequences for a part of the fungal taxa, it is difficult to clearly separate the many genera in this group using molecular data; therefore, a broad concept of *Hyphodontia* s.l. was accepted (Yurchenko and Wu 2016; Riebesehl and Langer 2017; Wang and Chen 2017; Riebesehl et al. 2019). On the basis of the nuclear DNA sequence data, six well-distinguished clades—*Lagarobasidium* clade, *Kneiffiella*-*Alutaceodontia* clade, *Hyphodontia* clade, *Hastodontia* clade, *Xylodon*-*Lyomyces*, *Rogersella* clade, and *Xylodon*-*Schizopora*-*Palifer* clade—were included based on the phylogenetical studies for *Hyphodontia* s.l., in which the genera *Xylodon* and *Lyomyces* were nested within the *Xylodon*-*Lyomyces*-*Rogersella* clade and *Xylodon*-*Schizopora*-*Palifer* clade, respectively (Yurchenko and Wu 2013). Riebesehl and Langer (2017) revealed that *Hyphodontia* s.l. was divided into six genera, viz., *Hastodontia* (Parmasto) Hjortstam & Ryvarden, *Hyphodontia* J. Erikss, *Kneiffiella*, *Lagarobasidium* Jülich, *Lyomyces*, and *Xylodon*, in which 35 new combinations were proposed, including fourteen *Lyomyces* species. Yurchenko et al. (2017) clarified the *Lyomycessambuci* complex on the basis of the sequences of the internal transcribed spacer (ITS) and the nuclear large subunit (nLSU) ribosomal DNA gene, in which four new species were described. To confirm the taxonomic relationship of *Xylodon* species, Viner et al. (2018) proposed two genera, *Lagarobasidium* and *Xylodon*, which should be synonymous based on molecular data from the ITS and nLSU regions, and in which the three species were combined into *Xylodon*. Riebesehl et al. (2019) clarified the generic concept and their phylogenetic reconstruction of *Lyomyces*, in which *L.sambuci* was sister to *L.crustosus* (Pers.) P. Karst. Based on a combination of morphological and molecular evidence, the wood-inhabiting fungal diversity within the family Schizoporaceae of the order Hymenochaetales were analyzed, including *Lyomycesfissuratus* C.L. Zhao, *L.fumosus* C.L. Zhao, *L.niveus* C.L. Zhao and *L.ochraceoalbus* C.L. Zhao. (Luo et al. 2021b, 2021c). Viner et al. (2022) described three new species from Africa as *Xylodonangustisporus* Viner & Ryvarden, *X.dissiliens* Viner & Ryvarden, and *X.laxiusculus* Viner & Ryvarden, based on the morphological descriptions and molecular analyses that they conducted. A phylogenetic and taxonomic study on *Xylodon* showed that three new species of *Xylodon* were from southern China; in addition, it was also found that it enriched the fungal diversity of these areas (Qu et al. 2022).

During investigations on the wood-inhabiting fungi in the Yunnan–Guizhou Plateau of China, samples representing five additional species belonging to genera *Lyomyces* and *Xylodon* were collected. To clarify the placement and relationships of the five species, we carried out a phylogenetic and taxonomic study on *Lyomyces* and *Xylodon*, based on the ITS and nLSU sequences.

## ﻿Materials and methods

### ﻿Morphology

The studied specimens were deposited at the Herbarium of Southwest Forestry University (SWFC), Kunming, Yunnan Province, P.R. China. Macromorphological descriptions are based on field notes and photos captured in the field and lab. Color terminology follows Petersen (Petersen 1996). Micromorphological data were obtained from the dried specimens when observed under a light microscope following Dai (2012). The following abbreviations are used: KOH = 5% potassium hydroxide water solution, CB = Cotton Blue, CB– = acyanophilous, IKI = Melzer’s Reagent, IKI– = both inamyloid and indextrinoid, L = mean spore length (arithmetic average for all spores), W = mean spore width (arithmetic average for all spores), Q = variation in the L/W ratios between the specimens studied and n = a/b (number of spores (a) measured from given number (b) of specimens).

### ﻿Molecular phylogeny

The CTAB rapid plant genome extraction kit-DN14 (Aidlab Biotechnologies Co., Ltd, Beijing) was used to obtain genomic DNA from the dried specimens following the manufacturer’s instructions (Zhao and Wu 2017). The nuclear ribosomal ITS region was amplified with the primers ITS5 and ITS4 (White et al. 1990). The nuclear ribosomal LSU gene was amplified with the primers LR0R and LR7 (Vilgalys and Hester 1990; Rehner and Samuels 1994). The PCR procedure for ITS and nLSU followed previous study (Zhao and Wu 2017). All newly-generated sequences were deposited in NCBI GenBank (Table 1).

**Table 1.** List of species, specimens and GenBank accession numbers of sequences used in this study.

Species name	Specimen No.	GenBank accession No.		Country	References
		*ITS*	*nLSU*		
*Fasciodontiabrasiliensis*	MSK-F 7245a	MK575201	MK598734	Brazil	Yurchenko et al. 2020
*F.bugellensis*	KAS-FD 10705a	MK575203	MK598735	France	Yurchenko et al. 2020
	MSK-F 7353	MK575205	MK598736	Belarus	Yurchenko et al. 2020
*F.yunnanensis*	CLZhao 6280	MK811275	MZ146327	China	Luo and Zhao 2021
	CLZhao 6385	MK811277	–	China	Luo and Zhao 2021
*Hastodontiahalonata*	HHB-17058	MK575207	MK598738	Mexico	Yurchenko et al. 2020
*Hymenochaeteochromarginata*	He 47	KU978861	JQ279666	China	Unpublished
*H.rubiginosa*	He 458	JQ279580	–	China	He and Li 2013
*Hyphodontiaarguta*	KHL 11938 (GB)	EU118632	EU118633	Sweden	Larsson 2007
*H.pallidula*	GEL 2097	DQ340317	DQ340372	Germany	Unpublished
*H.zhixiangii*	LWZ 20160909-4	KY440396	–	Uzbekistan	Kan et al. 2017
	LWZ 20160909-9	KY440398	–	Uzbekistan	Kan et al. 2017
*Kneiffiellaeucalypticola*	LWZ20180515-9	MT319411	MT319143	Australia	Wang et al. 2021
*K.palmae*	GEL3456	DQ340333	DQ340369	China	Yurchenko et al. 2020
*K.subalutacea*	GEL2196	DQ340341	DQ340362	Norway	Yurchenko et al. 2020
***Lyomycesalbopulverulentus***	CLZhao 21478*	**OP730712**	**OP730724**	**China**	**Present study**
*L.allantosporus*	KAS-GEL4933	KY800401	–	France	Yurchenko et al. 2017
	FR-0249548	KY800397	–	La Réunion	Yurchenko et al. 2017
*L.bambusinus*	CLZhao 4831	MN945968	–	China	Chen and Zhao 2020
	CLZhao 4808	MN945970	–	China	Chen and Zhao 2020
	CLZhao 4831	MN945968	–	China	Chen and Zhao 2020
*L.cremeus*	CLZhao 4138	MN945974	–	China	Chen and Zhao 2020
	CLZhao 8295	MN945972	–	China	Chen and Zhao 2020
*L.crustosus*	TASM:YG G39	MF382993	–	Uzbekistan	Gafforov et al. 2017
	UC2022841	KP814310	–	USA	Unpublished
*L.densiusculus*	Ryvarden 44818	OK273853	–	Uganda	Viner et al. 2022
*L.elaeidicola*	LWZ20180411-20	MT319458	–	Malaysia	Wang et al. 2021
	LWZ20180411-19	MT319457	–	Malaysia	Wang et al. 2021
*L.erastii*	TASM:YG 022	MF382992	–	Uzbekistan	Gafforov et al. 2017
	23cSAMHYP	JX857800	–	Spain	Unpublished
*L.fimbriatus*	Wu910620-7	MK575209	–	China	Yurchenko et al. 2020
	Wu911204-4	MK575210	–	China	Yurchenko et al. 2020
*L.fissuratus*	CLZhao 4352	MW713742	–	China	Luo et al. 2021a
	CLZhao 4291	MW713738	–	China	Luo et al. 2021a
*L.fumosus*	CLZhao 8188	MW713744	–	China	Luo et al. 2021a
*L.gatesiae*	LWZ20180515-3	MT319447	–	Australia	Wang et al. 2021
	LWZ20180515-32	MT319448	–	Australia	Wang et al. 2021
*L.griseliniae*	KHL 12971 (GB)	DQ873651	–	Costa Rica	Larsson et al. 2006
*L.juniperi*	FR-0261086	KY081799	–	La Réunion	Riebesehl and Langer 2017
*L.leptocystidiatus*	LWZ20170818-1	MT326514	–	China	Wang et al. 2021
	LWZ20170818-2	MT326513	–	China	Wang et al. 2021
*L.macrosporus*	CLZhao 4516	MN945977	–	China	Chen and Zhao 2020
*L.mascarensis*	KAS-GEL4833	KY800399	–	La Réunion	Yurchenko et al. 2020
	KAS-GEL4908	KY800400	–	La Réunion	Yurchenko et al. 2020
*L.microfasciculatus*	CLZhao 5109	MN954311	–	China	Chen and Zhao 2020
*L.niveus*	CLZhao 6431	MZ262541	MZ262526	China	Luo et al. 2021b
	CLZhao 6442	MZ262542	MZ262527	China	Luo et al. 2021b
*L.ochraceoalbus*	CLZhao 4385	MZ262535	MZ262521	China	Luo et al. 2021b
	CLZhao 4725	MZ262536	MZ262522	China	Luo et al. 2021b
*L.organensis*	MSK7247	KY800403	–	Brazil	Yurchenko et al. 2017
*L.orientalis*	GEL3376	DQ340325	–	China	Yurchenko et al. 2017
*L.pruni*	GEL2327	DQ340312	–	Germany	Larsson et al. 2006
	Ryberg 021018 (GB)	DQ873624	–	Sweden	Larsson et al. 2006
*L.sambuci*	KAS-JR7	KY800402	KY795966	Germany	Yurchenko et al. 2017
	83SAMHYP	JX857721	–	USA	Yurchenko et al. 2017
*L.vietnamensis*	TNM F9073	JX175044	–	Vietnam	Yurchenko et al. 2013
*L.wuliangshanensis*	CLZhao 4108	MN945980	–	China	Chen and Zhao 2020
	CLZhao 4167	MN945979	–	China	Chen and Zhao 2020
***L.yunnanensis***	**CLZhao 2463**	**OP730711**	**OP730723**	**China**	**Present study**
	**CLZhao 9375**	**OP730710**	–	**China**	**Present study**
	CLZhao 10041*	**OP730709**	–	**China**	**Present study**
*Xylodonacystidiatus*	LWZ20180514-9	MT319474	–	Australia	Wang et al. 2021
*X.apacheriensis*	Wu 0910-58	KX857797	–	China	Chen et al. 2017
*X.aspera*	KHL 8530	AY463427	–	Sweden	Larsson et al. 2004
*X.astrocystidiata*	Wu 9211-71	JN129972	–	China	Yurchenko and Wu 2014
*X.attenuatus*	Spirin 8775	MH324476	–	America	Viner et al. 2018
*X.australis*	LWZ20180509-8	MT319503	–	China	Wang et al. 2021
*X.bambusinus*	CLZhao 9174	MW394657	–	China	Ma and Zhao 2021
*X.borealis*	JS26064	AY463429	–	Norway	Larsson et al. 2004
*X.brevisetus*	JS17863	AY463428	–	Norway	Larsson et al. 2004
*X.crystalliger*	LWZ20170816-33	MT319521	–	China	Wang et al. 2021
*X.cystidiatus*	FR-0249200	MH880195	MH884896	Réunion	Riebesehl et al. 2019
*X.damansaraensis*	LWZ20180417-23	MT319499	–	Malaysia	Wang et al. 2021
***X.daweishanensis***	CLZhao 18357*	**OP730715**	–	**China**	**Present study**
	**CLZhao 18425**	**OP730716**	–	**China**	**Present study**
	**CLZhao 18446**	**OP730717**	**OP730725**	**China**	**Present study**
	**CLZhao 18458**	**OP730718**	**OP730726**	**China**	**Present study**
	**CLZhao 18492**	**OP730719**	**OP730727**	**China**	**Present study**
*X.detriticus*	Zíbarová 30.10.17	MH320793	–	Czech Republic	Viner et al. 2018
*X.filicinus*	MSK-F 12869	MH880199	NG067836	China	Riebesehl et al. 2019
***X.fissuratus***	CLZhao 9407*	**OP730714**	–	**China**	**Present study**
	**CLZhao 7007**	**OP730713**	–	**China**	**Present study**
*X.flaviporus*	FR-0249797	MH880201	–	Réunion	Riebesehl et al. 2019
*X.flocculosus*	CLZhao 18342	MW980776	–	China	Qu and Zhao 2022
*X.follis*	FR-0249814	MH880204	–	Réunion	Riebesehl et al. 2019
*X.gossypinus*	CLZhao 8375	MZ663804	MZ663813	China	Luo et al. 2021
*X.grandineus*	CLZhao 16075	OM338091	–	China	Luo et al. 2022
	CLZhao 6425	OM338090	–	China	Luo et al. 2022
*X.hastifer*	K(M) 172400	NR166558	–	USA	Riebesehl and Langer 2017
*X.heterocystidiatus*	Wei 17-314	MT731753	–	China	Unpublished
*X.hyphodontinus*	KAS-GEL9222	MH880205	MH884903	Kenya	Riebesehl et al. 2019
*X.kunmingensis*	TUB-FO 42565	MH880198	–	China	Riebesehl et al. 2019
*X.lacerates*	CLZhao 9892	OL619258	–	China	Qu et al. 2022
*X.lagenicystidiatus*	LWZ20180513-16	MT319634	–	Australia	Wang et al. 2021
*X.lenis*	Wu890714-3	KY081802	–	China	Riebesehl and Langer 2017
*X.macrosporus*	CLZhao 10226	MZ663809	MZ663817	China	Luo et al. 2021
*X.mollissimus*	LWZ 20160318-3	KY007517	–	China	Wang et al. 2021
*X.montanus*	CLZhao 8179	OL619260	–	China	Qu et al. 2022
*X.nespori*	LWZ20180921-35	MT319655	MT319238	China	Wang et al. 2021
*X.niemelaei*	LWZ20150707-13	MT319630	–	China	Wang et al. 2021
*X.nongravis*	GC 1412-22	KX857801	–	China	Chen et al. 2017
*X.nothofagi*	ICMP 13842	AF145583	–	China	Paulus et al. 2000
*X.ovisporus*	LWZ20170815-31	MT319666	–	China	Wang et al. 2021
*X.papillosa*	CBS:114.71	MH860026	–	Netherlands	Vu et al. 2019
*X.paradoxus*	Dai14983	MT319519	–	China	Wang et al. 2021
*X.pruinosus*	Spirin 2877	MH332700	–	Estonia	Viner et al. 2018
*X.pseudolanatus*	CFMR FP-150922	MH880220	–	Belize	Riebesehl et al. 2019
*X.pseudotropicus*	Dai16167	MT319509	–	China	Wang et al. 2021
***X.puerensis***	CLZhao 8142*	**OP730720**	**OP730728**	**China**	**Present study**
	**CLZhao 8639**	**OP730721**	**OP730729**	**China**	**Present study**
*X.punctus*	CLZhao 17691	OM338092	–	China	Luo et al. 2022
*X.quercinus*	Larsson 11076 (GB)	KT361633	–	Sweden	Larsson et al. 2004
*X.ramicida*	Spirin 7664	NR138013	–	usa	Unpublished
*X.rhododendricola*	LWZ20180513-9	MT319621	–	Australia	Wang et al. 2021
*X.rimosissima*	Ryberg 021031 (GB)	DQ873627	–	Sweden	Larsson et al. 2006
*X.serpentiformis*	LWZ20170816-15	MT319673	–	China	Wang et al. 2021
*X.sinensis*	CLZhao 11120	MZ663811	–	China	Luo et al. 2021
*X.spathulatus*	LWZ20180804-10	MT319646	–	China	Wang et al. 2021
*X.subclavatus*	TUB-FO 42167	MH880232	–	China	Riebesehl et al. 2019
*X.subflaviporus*	Wu 0809-76	KX857803	–	China	Chen et al. 2017
*X.subserpentiformis*	LWZ20180512-16	MT319486	–	Australia	Wang et al. 2021
*X.subtropicus*	LWZ20180510-24	MT319541	–	China	Wang et al. 2021
*X.taiwanianus*	CBS:125875	MH864080	–	Netherlands	Vu et al. 2019
*X.tropicus*	CLZhao 3351	OL619261	OL619269	China	Qu et al. 2022
*X.ussuriensis*	KUN 1989	NR166241	–	USA	Unpublished
*X.verecundus*	KHL 12261 (GB)	DQ873642	–	Sweden	Larsson et al. 2006
*X.victoriensis*	LWZ20180510-29	MT319487	–	Australia	Wang et al. 2021
*X.wenshanensis*	CLZhao 10790	OM338095	–	China	Luo et al. 2022
	CLZhao 15729	OM338097	OM338104	China	Luo et al. 2022
*X.xinpingensis*	CLZhao 11224	MW394662	MW394654	China	Luo et al. 2022
*X.yarraensis*	LWZ20180510-5	MT319639	–	Australia	Wang et al. 2021
*X.yunnanensis*	LWZ20180922-47	MT319660	–	China	Wang et al. 2021

* Indicates type materials.

**Figure 1.**
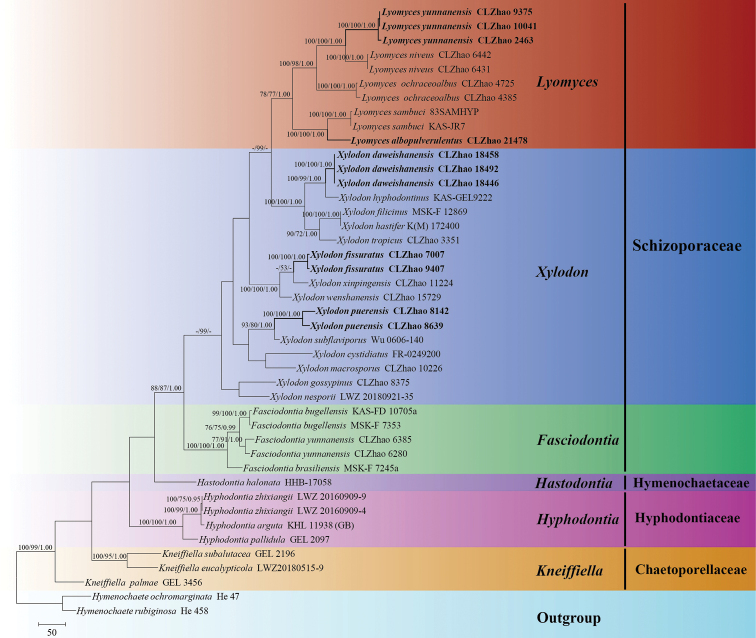
Maximum parsimony strict consensus tree illustrating the phylogeny of *Xylodon*, *Lyomyces* and related genera in the order Hymenochaetales based on ITS+nLSU sequences. Branches are labelled with maximum likelihood bootstrap values > 70%, parsimony bootstrap values > 50% and Bayesian posterior probabilities > 0.95, respectively.

**Figure 2.**
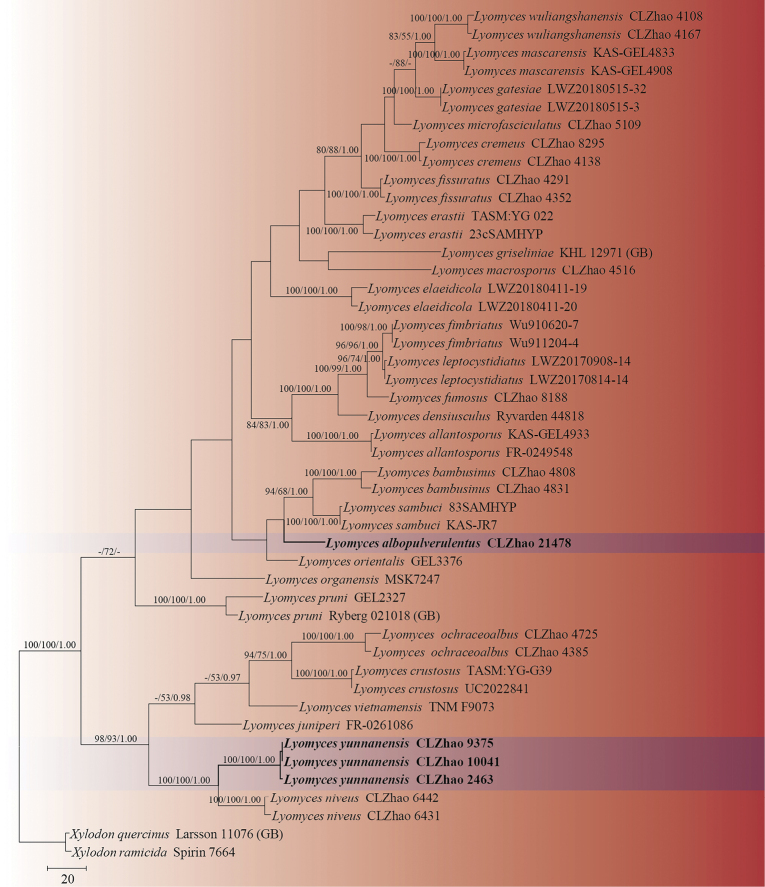
Maximum parsimony strict consensus tree illustrating the phylogeny of the two new species and related species in *Lyomyces*, based on ITS sequences. Branches are labelled with maximum likelihood bootstrap values > 70%, parsimony bootstrap values > 50% and Bayesian posterior probabilities > 0.95, respectively.

**Figure 3.**
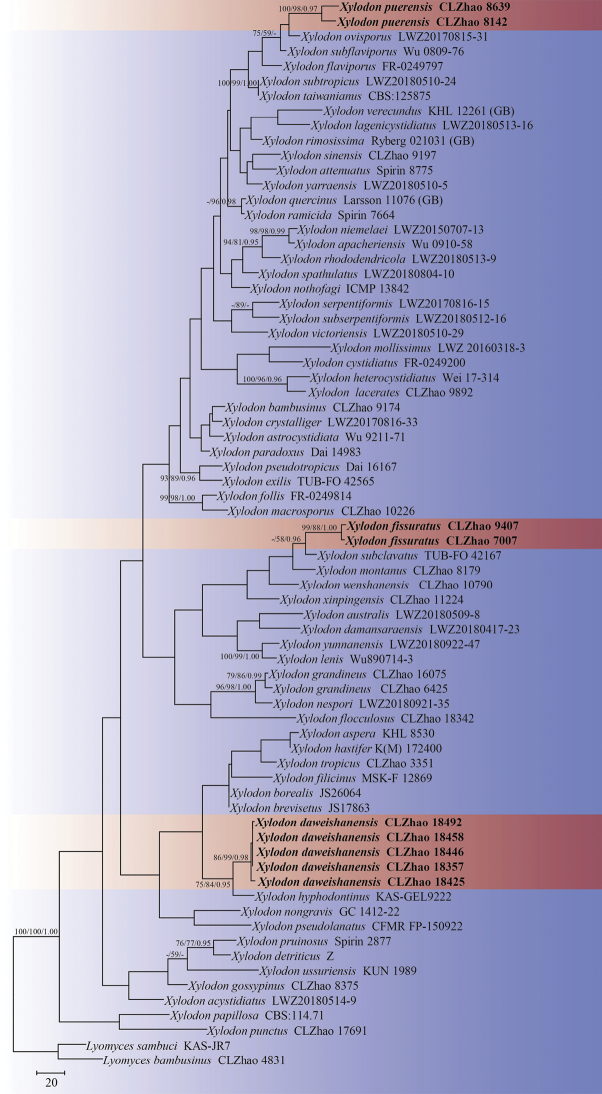
Maximum parsimony strict consensus tree illustrating the phylogeny of the three new species and related species in *Xylodon*, based on ITS sequences. Branches are labelled with maximum likelihood bootstrap values > 70%, parsimony bootstrap values > 50% and Bayesian posterior probabilities > 0.95, respectively.

The sequences were aligned in MAFFT version 7 (Katoh et al. 2019) using the G-INS-i strategy. The alignment was adjusted manually using AliView version 1.27 (Larsson 2014). Each dataset was aligned separately at first and then the ITS and nLSU regions were combined with Mesquite version 3.51. The combined dataset was deposited in TreeBASE (submission ID 29868). Sequences of *Hymenochaeteochromarginata* P.H.B. Talbot and *H.rubiginosa* (Dicks.) Lév. retrieved from GenBank were used as an outgroup in the ITS+nLSU analysis (Fig. 1); Sequences of *Xylodonquercinus* and *X.ramicida* Spirin & Miettinen retrieved from GenBank were used as an outgroup in the ITS analysis (Fig. 2); *Lyomycesbambusinus* C.L. Zhao and *L.sambuci* were selected as outgroup (Fig. 3) as inspired by a previous study (Luo et al. 2021c).

Maximum parsimony analysis in PAUP* version 4.0a169 (http://phylosolutions.com/paup-test/) was applied to ITS and the combined ITS+nLSU datasets following a previous study (Zhao and Wu 2017). All characters were equally weighted and gaps were treated as missing data. Trees were inferred using the heuristic search option with TBR branch swapping and 1,000 random sequence additions. Max-trees were set to 5,000, branches of zero length were collapsed and all parsimonious trees were saved. Clade robustness was assessed using bootstrap (BT) analysis with 1,000 pseudo replicates (Felsenstein 1985). Descriptive tree statistics – tree length (TL), composite consistency index (CI), composite retention index (RI), composite rescaled consistency index (RC) and composite homoplasy index (HI) – were calculated for each maximum parsimonious tree generated. The combined dataset was also analysed using Maximum Likelihood (ML) in RAxML-HPC2 through the CIPRES Science Gateway (Miller et al. 2012). Branch support (BS) for the ML analysis was determined by 1000 bootstrap pseudoreplicates.

MrModeltest 2.3 (Nylander 2004) was used to determine the best-ﬁt evolution model for each dataset for the purposes of Bayesian inference (BI). which was performed using MrBayes 3.2.7a with a GTR+I+G model of DNA substitution and a gamma distribution rate variation across sites (Ronquist et al. 2012). A total of four Markov chains were run for two runs from random starting trees for 8 million generations for ITS+nLSU (Fig. 1); 0.5 million generations for ITS (Fig. 2) and 9.5 million generations for ITS (Fig. 3) with trees and parameters sampled every 1,000 generations. The ﬁrst quarter of all of the generations were discarded as burn-ins. A majority rule consensus tree was computed from the remaining trees. Branches were considered as significantly supported if they received a maximum likelihood bootstrap support value (BS) of > 70%, a maximum parsimony bootstrap support value (BT) of > 70% or a Bayesian posterior probability (BPP) of > 0.95.

## ﻿Results

### ﻿Molecular phylogeny

The ITS+nLSU dataset (Fig. 1) comprised sequences from 43 fungal specimens representing 31 taxa. The dataset had an aligned length of 2,100 characters, of which 1,323 characters were constant, 156 were variable and parsimony-uninformative and 621 (35%) were parsimony-informative. Maximum parsimony analysis yielded 1 equally parsimonious tree (TL = 2,867, CI = 0.4423, HI = 0.5577, RI = 0.6488 and RC = 0.2869). The best model of nucleotide evolution for the ITS+nLSU dataset estimated and applied in the Bayesian analysis was found to be GTR+I+G. Bayesian analysis and ML analysis resulted in a similar topology as in the MP analysis. The Bayesian analysis had an average standard deviation of split frequencies = 0.008603 (BI) and the effective sample size (ESS) across the two runs is double the average ESS (avg. ESS) = 1,623. The phylogram based on the ITS+nLSU rDNA gene regions (Fig. 1) include six genera within Schizoporaceae (Hymenochaetales), which are *Fasciodontia*, *Hastodontia*, *Hyphodontia*, *Kneifiella*, *Lyomyces*, and *Xylodon*—in which five new species were grouped into the genera *Lyomyces* and *Xylodon*.

The ITS dataset (Fig. 2) comprised sequences from 47 fungal specimens representing 29 taxa. The dataset had an aligned length of 661 characters, of which 316 characters were constant, 53 were variable and parsimony-uninformative and 292 (35%) were parsimony-informative. Maximum parsimony analysis yielded 1 equally parsimonious tree (TL = 1,371, CI = 0.4136, HI = 0.5864, RI = 0.6984 and RC = 0.2888). The best model of nucleotide evolution for the ITS dataset estimated and applied in the Bayesian analysis was found to be GTR+I+G. Bayesian analysis and ML analysis resulted in a similar topology as in the MP analysis. The Bayesian analysis had an average standard deviation of split frequencies = 0.006564 (BI) and the effective sample size (ESS) across the two runs is double the average ESS (avg. ESS) = 359. The phylogenetic tree (Fig. 2), inferred from the ITS sequences, highlighted that *L.albopulverulentus* formed a monophyletic lineage. It was then grouped closely with *L.bambusinus*, *L.orientalis* Riebesehl, Yurch. & Langer, and *L.sambuci*. In addition, *L.yunnanensis* was found to be the sister to *L.niveus* with strong supports.

The ITS dataset (Fig. 3) comprised sequences from 72 fungal specimens representing 65 taxa. The dataset had an aligned length of 702 characters, of which 283 characters were constant, 96 were variable and parsimony-uninformative and 323 (35%) were parsimony-informative. Maximum parsimony analysis yielded 5,000 equally parsimonious trees (TL = 2,726, CI = 0.2748, HI = 0.7252, RI = 0.4280 and RC = 0.1176). The best model of nucleotide evolution for the ITS dataset estimated and applied in the Bayesian analysis was found to be GTR+I+G. Bayesian analysis and ML analysis resulted in a similar topology as in the MP analysis. The Bayesian analysis had an average standard deviation of split frequencies = 0.02518 (BI) and the effective sample size (ESS) across the two runs is double the average ESS (avg. ESS) = 1,440. The topology (Fig. 3), based on ITS sequences, revealed that *X.daweishanensis* was retrieved as a sister to *X.hyphodontinus* (Hjortstam & Ryvarden) Riebesehl, Yurchenko & G. Gruhn. Furthermore, *X.fissuratus* was grouped with four taxa: *X.montanus* C.L. Zhao; *X.subclavatus* (Yurchenko, H.X. Xiong & Sheng H. Wu) Riebesehl, Yurch. & Langer; *X.wenshanensis* K.Y. Luo & C.L. Zhao; and *X.xinpingensis* C.L. Zhao & X. Ma. Moreover, *X.puerensis* was clustered with *X.flaviporus* (Berk. & M.A. Curtis ex Cooke) Riebesehl & Langer, *X.ovisporus* (Corner) Riebesehl & Langer, *X.subflaviporus* C.C. Chen & Sheng H. Wu, *X.subtropicus* (C.C. Chen & Sheng H. Wu) C.C. Chen & Sheng H. Wu, and *X.taiwanianus* (Sheng H. Wu) Hjortstam & Ryvarden.

### ﻿Taxonomy

#### ﻿ Lyomyces albopulverulentus

Taxon classificationFungiCorticialesCorticiaceae

C.L. Zhao sp. nov.

C170B90E-BD02-5CF5-A433-DC4F6341C458

846525

Figs 4 , 5

##### Type material.

***Holotype*.** China. Yunnan Province, Lijiang, Lashihai Nature Reserve, 26°51'37"N, 100°8'14"E, altitude 2450 m a.s.l., on fallen angiosperm branch, leg. C.L. Zhao, 19 July 2021, CLZhao 21478 (SWFC).

##### Etymology.

*Albopulverulentus* (Lat.): referring to the white and pruinose hymenial surface.

##### Description.

Basidiomata annual, resupinate, adnate, brittle, without odor or taste when fresh, up to 12 cm long, 1.5 cm wide, and 150 µm thick. Hymenial surface pruinose, white when fresh and drying. Sterile margin indistinct, white, and up to 2 mm wide.

**Figure 4.**
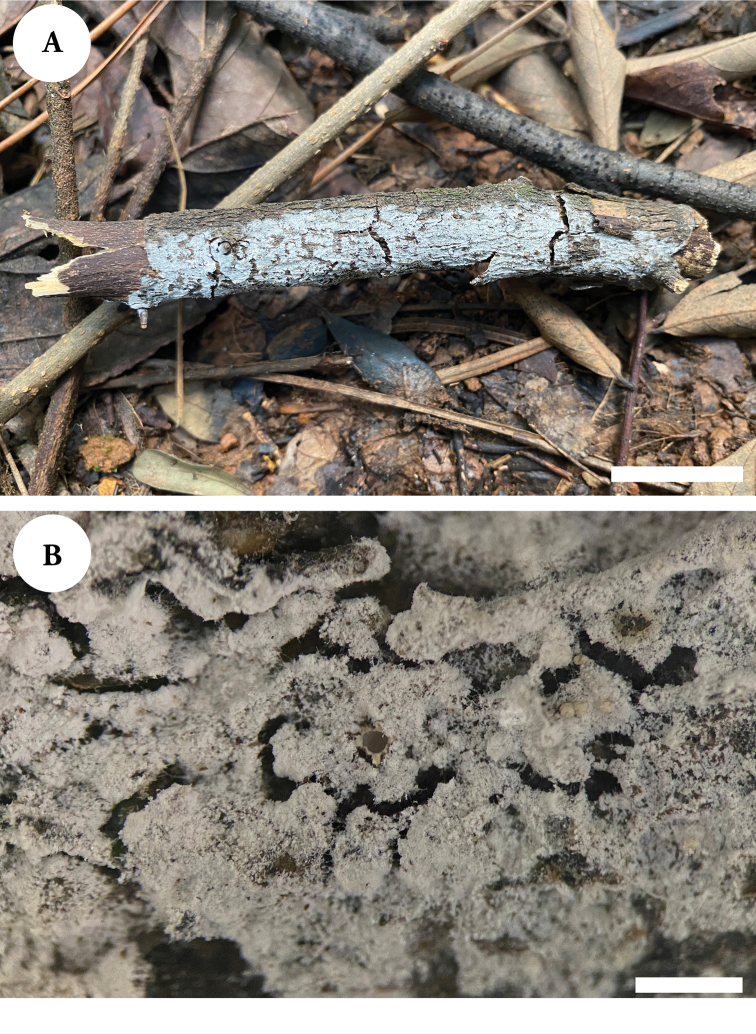
Basidiomata of *Lyomycesalbopulverulentus* (holotype). Scale bars: 2 cm (**A**); 1 mm (**B**).

Hyphal system monomitic, generative hyphae with clamp connections, colorless, thick-walled, frequently branched, interwoven, 3.5–5.5 µm in diameter; IKI–, CB–, tissues unchanged in KOH; subhymenial hyphae densely covered by crystals.

Cystidia capitate, colorless, thin-walled, smooth, slightly constricted at the neck, with a globose tip, 37–54 × 5–9 µm; basidia clavate, slightly sinuous, with four sterigmata and a basal clamp connection, 24.5–28.5 × 7–9 µm.

**Figure 5.**
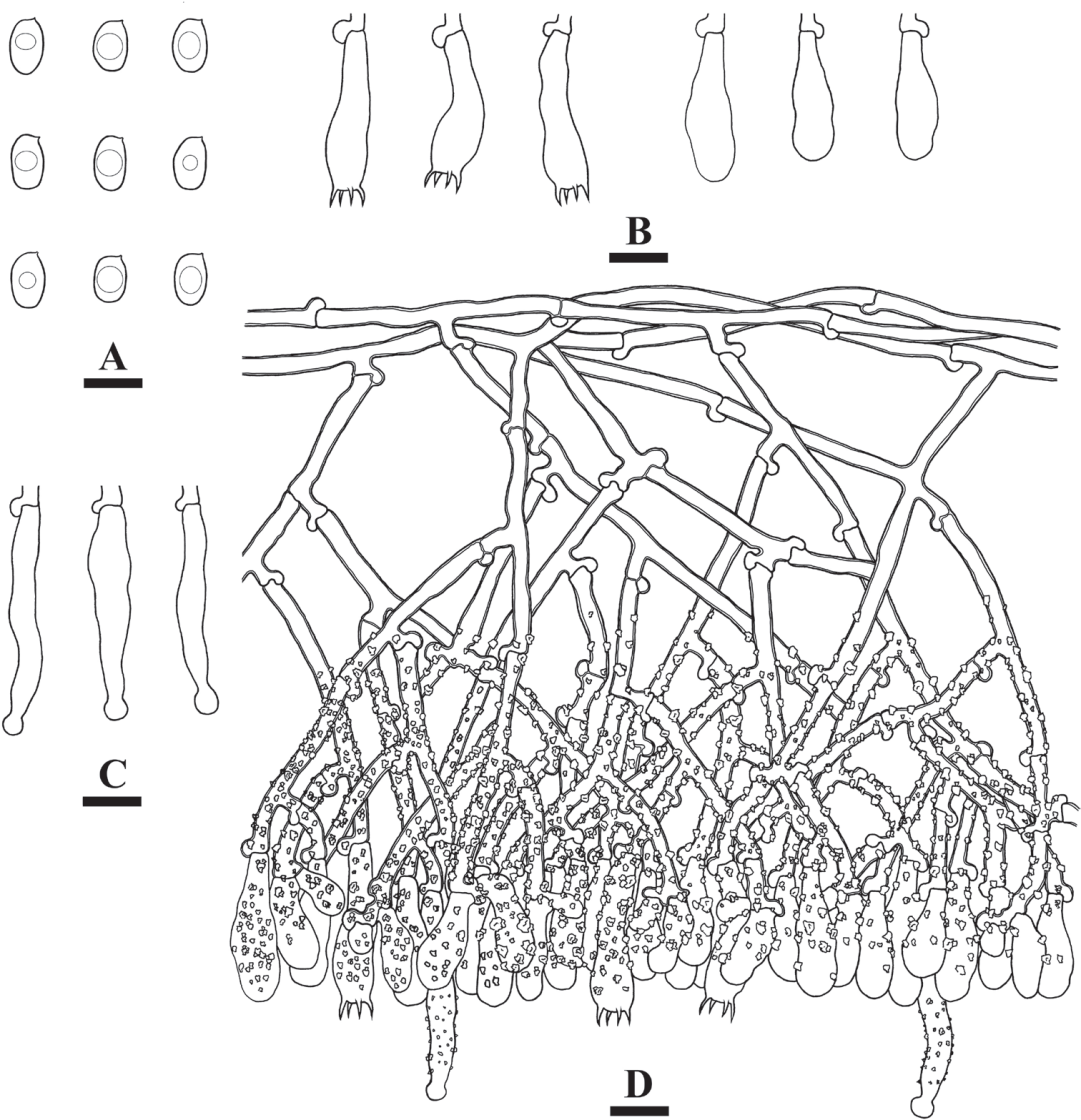
Microscopic structures of *Lyomycesalbopulverulentus* (holotype) **A** basidiospores **B** basidia and basidioles **C** capitate cystidia **D** a section of hymenium. Scale bars: 10 µm (**A–D**).

Basidiospores ellipsoid, colorless, thin-walled, smooth, IKI–, CB–, (7.5–)8–10.5(–11) × (5–)5.5–7 µm, L =9.12 µm, W = 6 µm, Q = 1.52 (n = 30/1).

##### Additional specimen examined

**(paratype).** China. Yunnan Province, Yuxi, Xinping County, the Ancient Tea Horse Road, 23°57'10"N, 101°30'41"E, altitude 2,600 m a.s.l., on fallen angiosperm branch, leg. C.L. Zhao, 13 January 2018, CLZhao 5234 (SWFC).

#### ﻿ Lyomyces yunnanensis

Taxon classificationFungiCorticialesCorticiaceae

C.L. Zhao sp. nov.

8BF809ED-DD39-50BA-8F3D-CAC7EA44A101

846527

Figs 6 , 7

##### Type material.

***Holotype*.** China. Yunnan Province, Dali, Nanjian County, Lingbaoshan, 24°46'2"N, 100°30'26"E, altitude 2350 m a.s.l., on fallen angiosperm branch, leg. C.L. Zhao, 9 January 2019, CLZhao 10041 (SWFC).

##### Etymology.

*Yunnanensis* (Lat.): referring to the locality (Yunnan Province) of the type specimen.

##### Description.

Basidiomata annual, resupinate, adnate, coriaceous when fresh, becoming farinaceous upon drying, without odor or taste when fresh, up to 15 cm long, 2.5 cm wide, and 150 µm thick. Hymenial surface grandinioid, cream to buff when fresh, and buff upon drying. Sterile margin indistinct, buff, and up to 1 mm wide.

**Figure 6.**
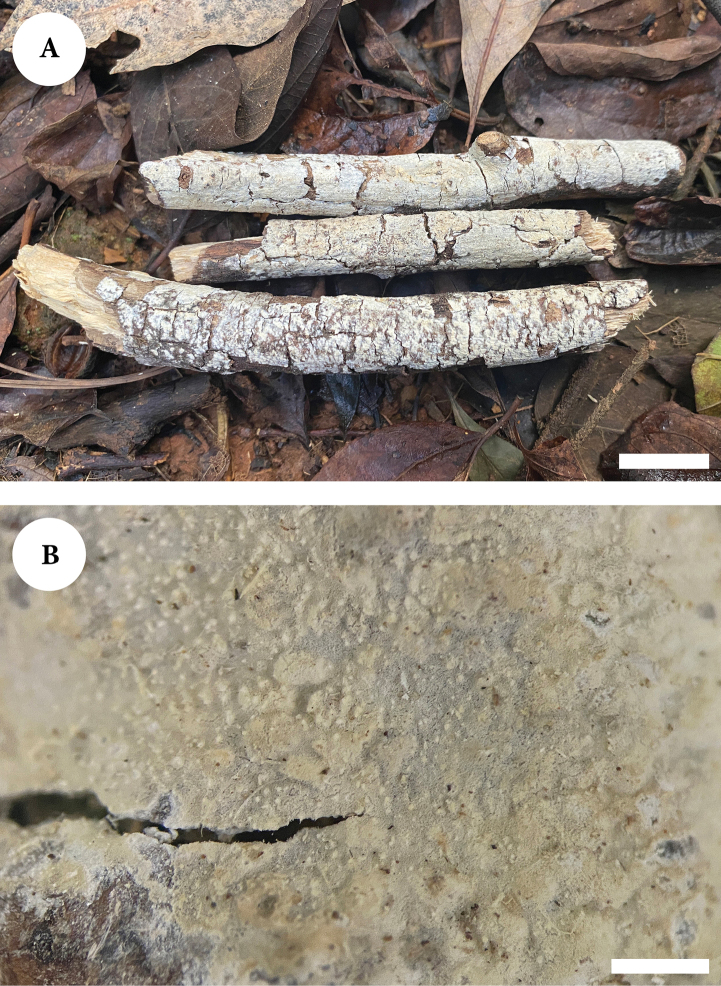
Basidiomata of *Lyomycesyunnanensis* (holotype). Scale bars: 2 cm (**A**); 1 mm (**B**).

Hyphal system monomitic, generative hyphae with clamp connections, colorless, thick-walled, frequently branched, interwoven, 2.5–3 µm in diameter; IKI–, CB–, tissues unchanged in KOH. Numerous crystals present among hyphae.

Cystidia of two types: (1) fusiform, tapering, colorless, thin-walled, 18–39 × 4–6 µm; (2) capitate cystidia, colorless, thin-walled, 16–23.5 × 3–5 µm; fusoid cystidioles present, colorless, thin-walled, 18–25 × 3–6 µm; basidia clavate, slightly sinuous, with four sterigmata and a basal clamp connection, 16.5–27 × 4–5.5 µm.

**Figure 7.**
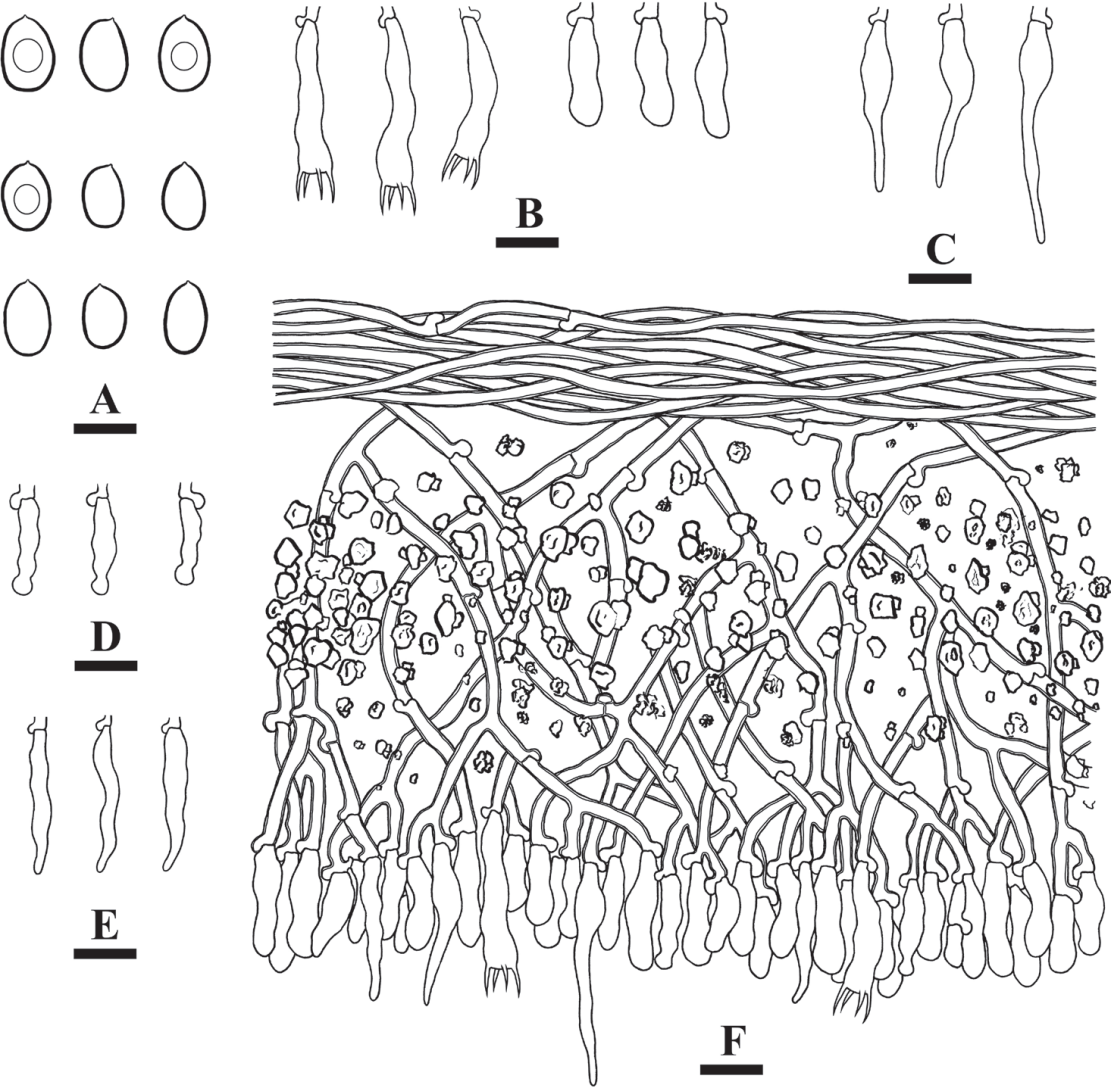
Microscopic structures of *Lyomycesyunnanensis* (holotype) **A** basidiospores **B** basidia and basidioles **C** tapering cystidia **D** capitate cystidia **E** fusoid cystidioles **F** a section of hymenium. Scale bars: 5 µm (**A**); 10 µm (**B–F**).

Basidiospores ellipsoid, colorless, thin-walled, smooth, IKI–, CB–, (4.5–)5–7 × 3–4.5 µm, L = 5.72 µm, W = 3.6 µm, Q = 1.54–1.65 (n = 90/3).

##### Additional specimens examined

**(paratypes).** China. Yunnan Province, Yuxi, Xinping County, Mopanshan National Forestry Park, 23°55'48"N, 101°59'22"E, altitude 2150 m a.s.l., on fallen angiosperm branch, leg. C.L. Zhao, 19 August 2017, CLZhao 2463 (SWFC); Puer, Jingdong County, the Forest of Pineapple, 24°21'32"N, 100°48'12"E, altitude 2110 m a.s.l., on fallen angiosperm branch, leg. C.L. Zhao, 4 January 2019, CLZhao 9375 (SWFC).

#### ﻿ Xylodon daweishanensis

Taxon classificationFungiHymenochaetalesSchizoporaceae

C.L. Zhao sp. nov.

5701FC42-658E-54B0-B599-7218CFC71868

846530

Figs 8 , 9

##### Type material.

***Holotype*.** China. Yunnan Province, Honghe, Pingbian County, Daweishan National Nature Reserve, 22°53'26"N, 103°35'37"E, altitude 1990 m a.s.l., on angiosperm trunk, leg. C.L. Zhao, 3 August 2019, CLZhao 18357 (SWFC).

##### Etymology.

*Daweishanensis* (Lat.): referring to the locality (Daweishan) of the type specimen.

##### Description.

Basidiomata annual, resupinate, adnate, without odor or taste when fresh, coriaceous, up to 10 cm long, 5 cm wide, and 150 µm thick. Hymenial surface odontioid, slightly buff when fresh, and buff upon drying. Margin sterile, slightly buff, and 1 mm wide.

Hyphal system monomitic, generative hyphae with clamp connections, colorless, thin to thick-walled, frequently branched, interwoven, 1.5–4 µm in diameter, IKI–, CB–, tissues unchanged in KOH.

**Figure 8.**
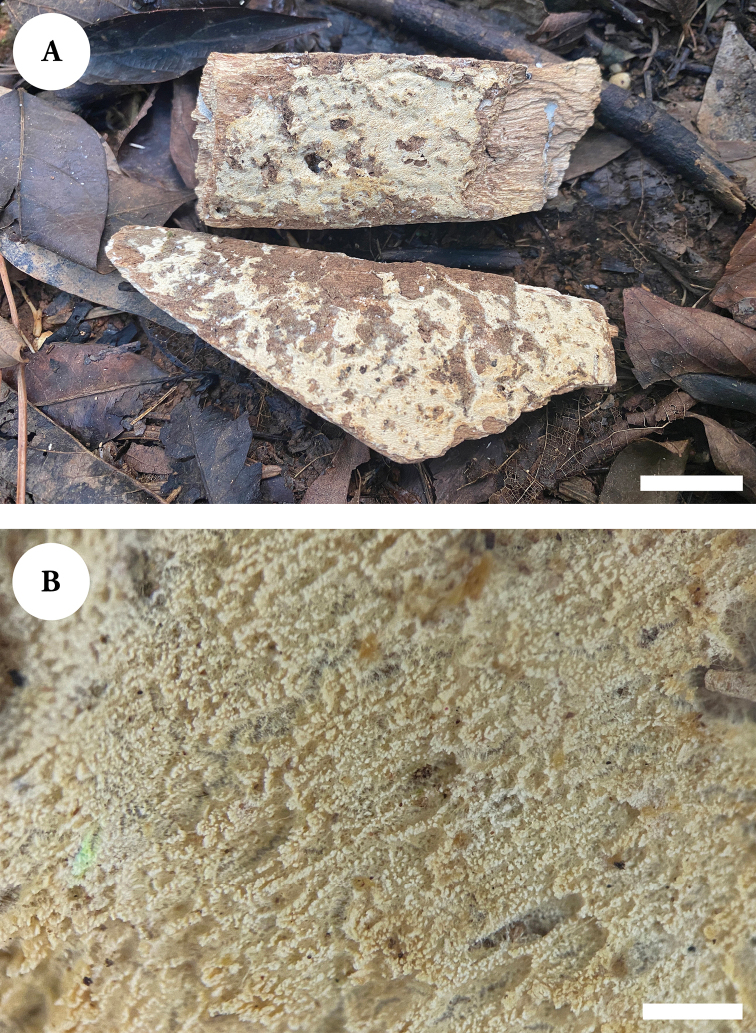
Basidiomata of *Xylodondaweishanensis* (holotype). Scale bars: 2 cm (**A**); 1 mm (**B**).

Cystidia of two types: (1) capitate cystidia thin-walled, smooth, slightly constricted at the neck, with a globose tip, 11–23.5 × 2.5–5 µm; (2) asterocystidia thin-walled, with the apical part encrusted, 11–26.5 × 2.5–4.5 µm; basidia clavate to subcylindrical, constricted, somewhat sinuous, with four sterigmata and a basal clamp connection, 11–15.5 × 2.5–4 µm.

**Figure 9.**
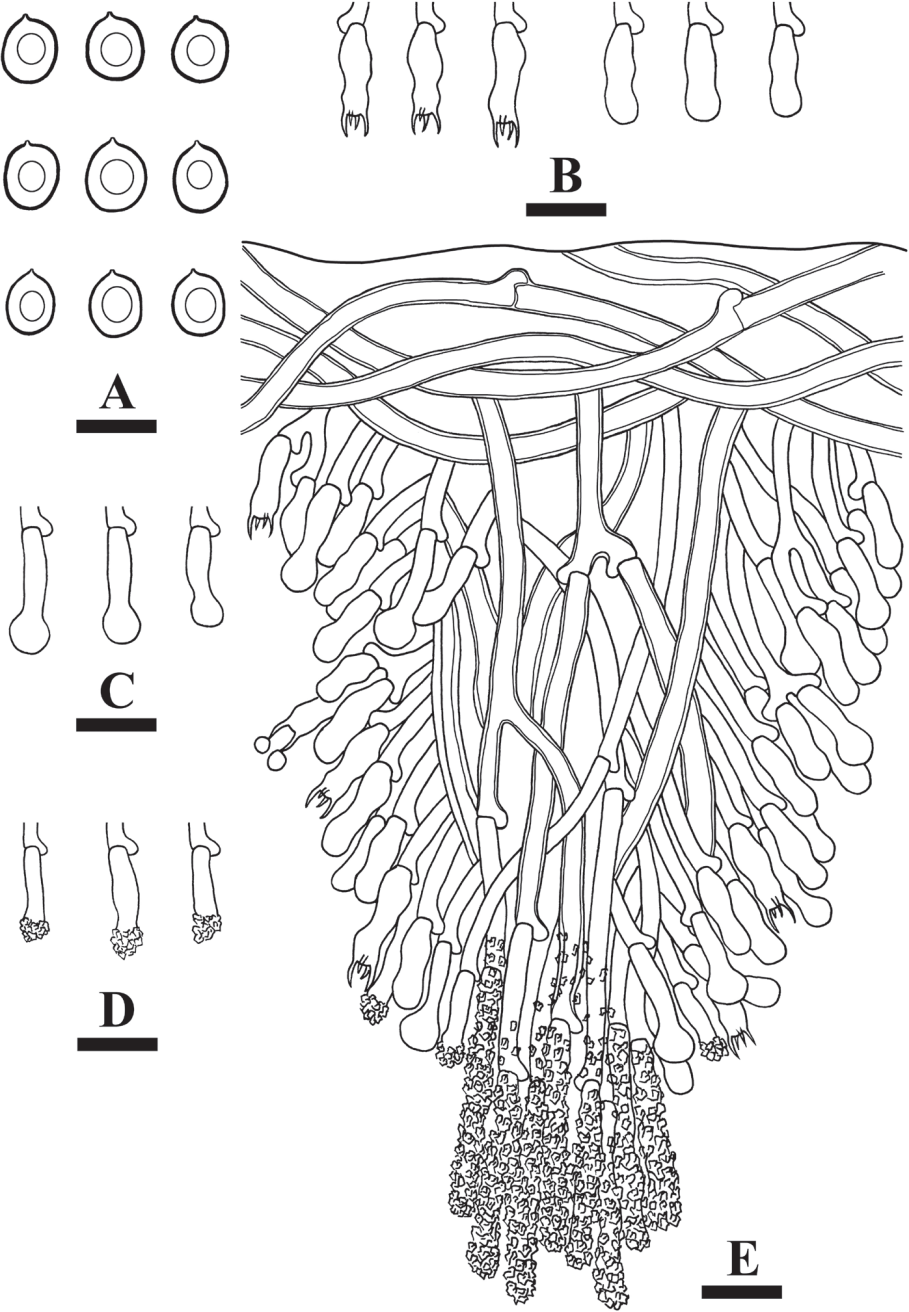
Microscopic structures of *Xylodondaweishanensis* (holotype) **A** basidiospores **B** basidia and basidioles **C** capitate cystidia **D** asterocystidia **E** a section of hymenium. Scale bars: 5 µm (**A**); 10 µm (**B–E**).

Basidiospores broad ellipsoid to subglobose, colorless, thin-walled, smooth, with oil drops, IKI–, CB–, 3–4 × 2.5–3.5(–4) µm, L = 3.51 µm, W = 3.14 µm, Q = 1.09–1.15 (n = 150/5).

##### Additional specimens examined

**(paratypes).** China. Yunnan Province, Honghe, Pingbian County, Daweishan National Nature Reserve, 22°53'26"N, 103°35'37"E, altitude 1990 m a.s.l., on angiosperm trunk, leg. C.L. Zhao, 3 August 2019, CLZhao 18425, CLZhao 18446, CLZhao 18458, and CLZhao 18492 (SWFC).

#### ﻿ Xylodon fissuratus

Taxon classificationFungiHymenochaetalesSchizoporaceae

C.L. Zhao sp. nov.

C081F893-D068-5A92-A8B6-6FF422B2ADC9

846532

Figs 10 , 11

##### Type material.

***Holotype*.** China. Yunnan Province, Puer, Jingdong County, the Forest of Pineapple, 24°21'32"N, 100°48'12"E, altitude 2110 m a.s.l., on fallen angiosperm branch, leg. C.L. Zhao, 4 January 2019, CLZhao 9407 (SWFC).

##### Etymology.

*Fissuratus* (Lat.): referring to the cracking hymenial surface.

##### Description.

Basidiomata annual, resupinate, adnate, coriaceous, without odor or taste when fresh, up to 12 cm long, 2.5 cm wide, and 150 µm thick. Hymenial surface grandinioid, and white when fresh, white to slightly cream on drying, cracking. Sterile margin indistinct, white, and up to 1 mm wide.

**Figure 10.**
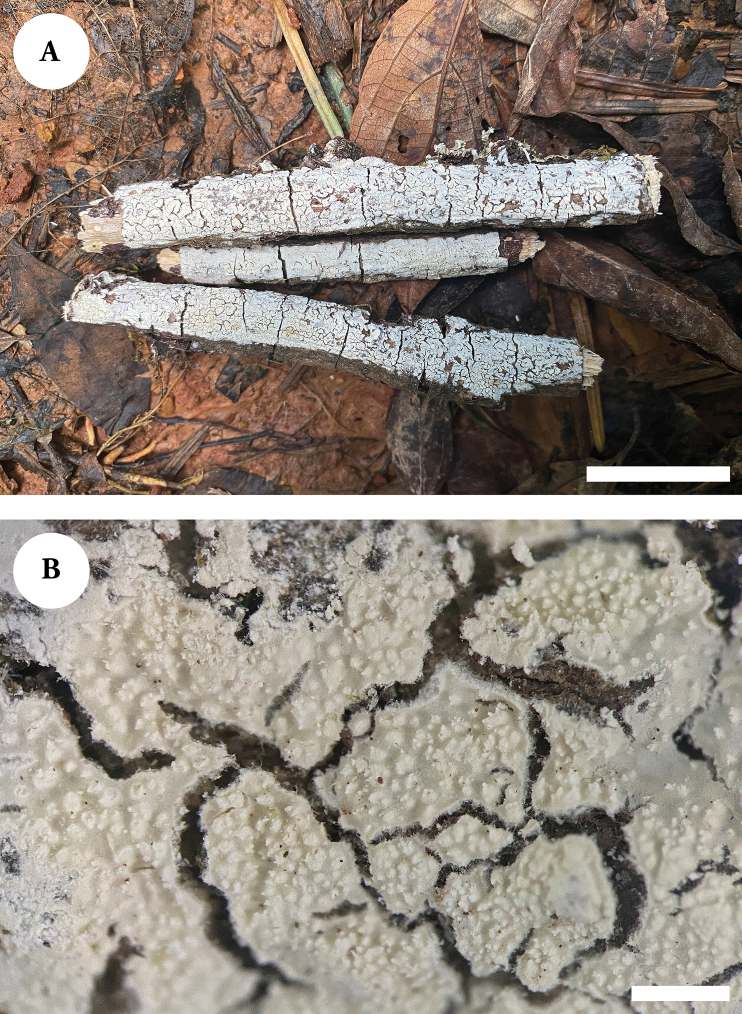
Basidiomata of *Xylodonfissuratus* (holotype). Scale bars: 2 cm (**A**); 1 mm (**B**).

Hyphal system monomitic, generative hyphae with clamp connections, colorless, thin-walled, frequently branched, interwoven, 2–3 µm in diameter; IKI–, CB–, tissues unchanged in KOH.

**Figure 11.**
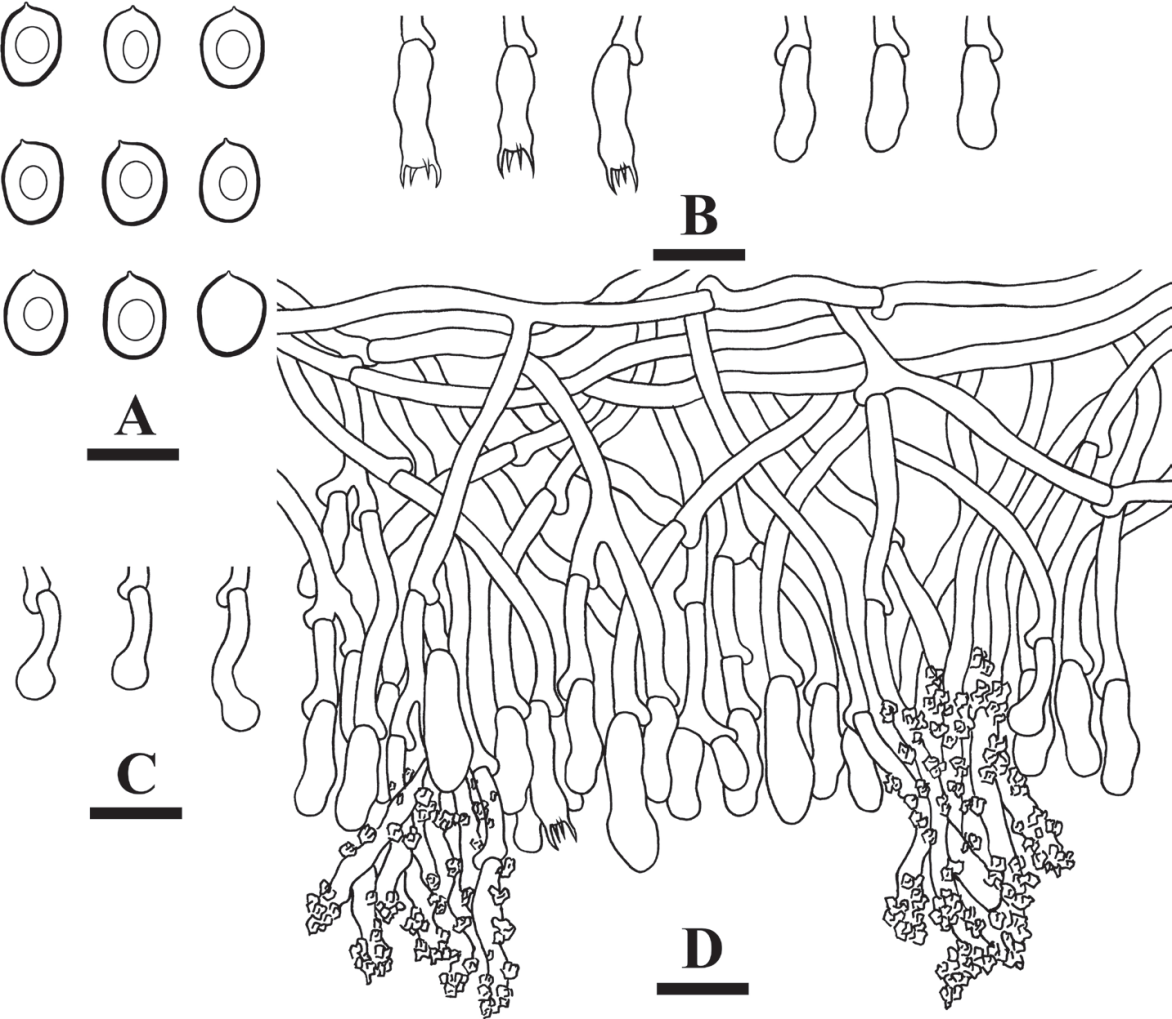
Microscopic structures of *Xylodonfissuratus* (holotype) **A** basidiospores **B** basidia and basidioles **C** capitate cystidia **D** a section of hymenium. Scale bars: 5 µm (**A**); 10 µm (**B–D**).

Cystidia capitate, thin-walled, smooth, slightly constricted at the neck, with a globose tip, 11.5–16.5 × 3–4.5 µm; basidia clavate to subcylindrical, slightly constricted in the middle to somewhat sinuous, with four sterigmata and a basal clamp connection, 10.5–16.5 × 2–4 µm.

Basidiospores ellipsoid, colorless, thin-walled, smooth, IKI–, CB–, 4–5 × 3–4 µm, L = 4.44 µm, W = 3.4 µm, Q = 1.3 (n = 30/1).

##### Additional specimen examined

**(paratype).** China. Yunnan Province, Chuxiong, Zixishan Forestry Park, 25°01'26"N, 101°24'37"E, altitude 2313 m a.s.l., on fallen angiosperm branch, leg. C.L. Zhao, 1 July 2018, CLZhao 7007 (SWFC).

#### ﻿ Xylodon puerensis

Taxon classificationFungiHymenochaetalesSchizoporaceae

C.L. Zhao sp. nov.

38732B62-69BE-51E7-A6F4-38A84D1FFC30

846533

Figs 12 , 13

##### Type material.

***Holotype*.** China. Yunnan Province, Puer, Zhenyuan County, Heping Town, Jinshan Virgin Forest Park, 23°56'21"N, 101°25'32"E, altitude 2240 m a.s.l., on fallen angiosperm branch, leg. C.L. Zhao, 21 August 2018, CLZhao 8142 (SWFC).

##### Etymology.

*Puerensis* (Lat.): referring to the locality (Yunnan Province) of the type specimen.

##### Description.

Basidiomata annual, resupinate, adnate, coriaceous, without odor or taste when fresh, up to 12 cm long, 5 cm wide, and 200 µm thick. Hymenial surface poroid, pores angular or slightly daedaleoid, 3–6 per mm, and cream when fresh, buff on drying. Sterile margin slightly buff, and up to 1 mm wide.

**Figure 12.**
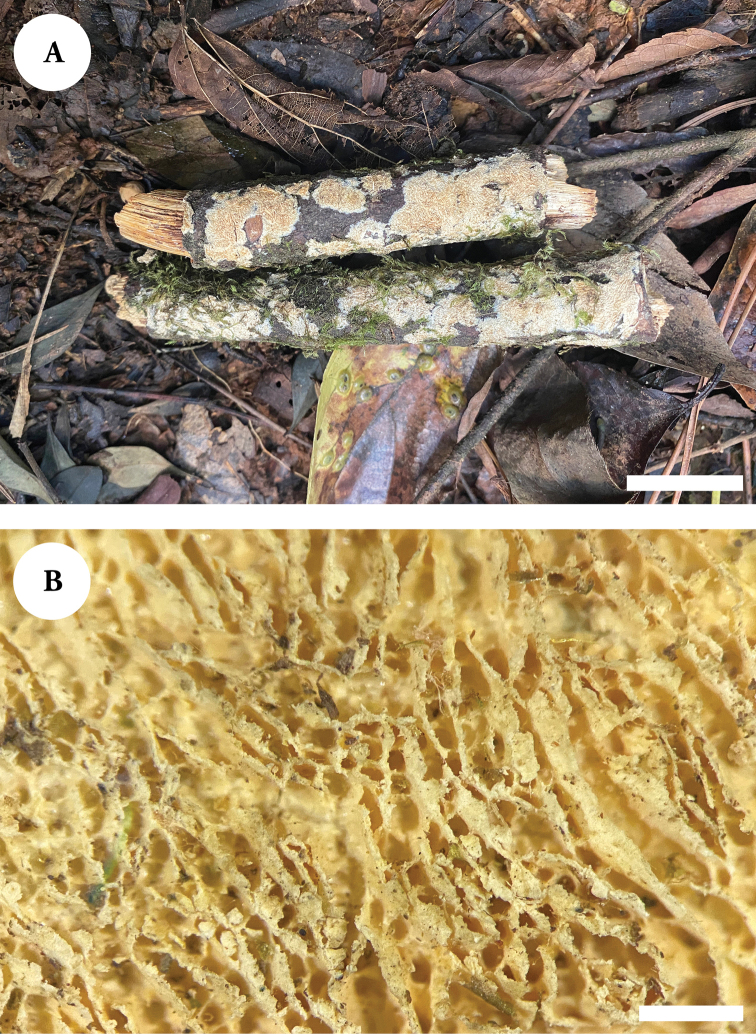
Basidiomata of *Xylodonpuerensis* (holotype). Scale bars: 2 cm (**A**); 1 mm (**B**).

Hyphal system monomitic, generative hyphae with clamps, colorless, thick-walled, frequently branched, interwoven, 2.5–4.5 µm in diameter; IKI–, CB–, tissues unchanged in KOH.

Cystidia of four types: (1) paraphysoid cystidia colorless, smooth, 12–20.5 × 3–5 µm; (2) astrocystidia colorless, thin-walled, smooth, 9–11 × 3.5–5.5 µm; (3) capitate cystidia, colorless, thin-walled, smooth, embedded, 22–29.5 × 6.5–12 µm; (4) septocystidia, thin-walled, smooth, with the apical part encrusted, 32–51 × 3.5–6 µm; basidia clavate to subcylindrical, slightly sinuous or distinctly sinuous, with four sterigmata and a basal clamp connection, 14.5–20 × 5–7 µm.

**Figure 13.**
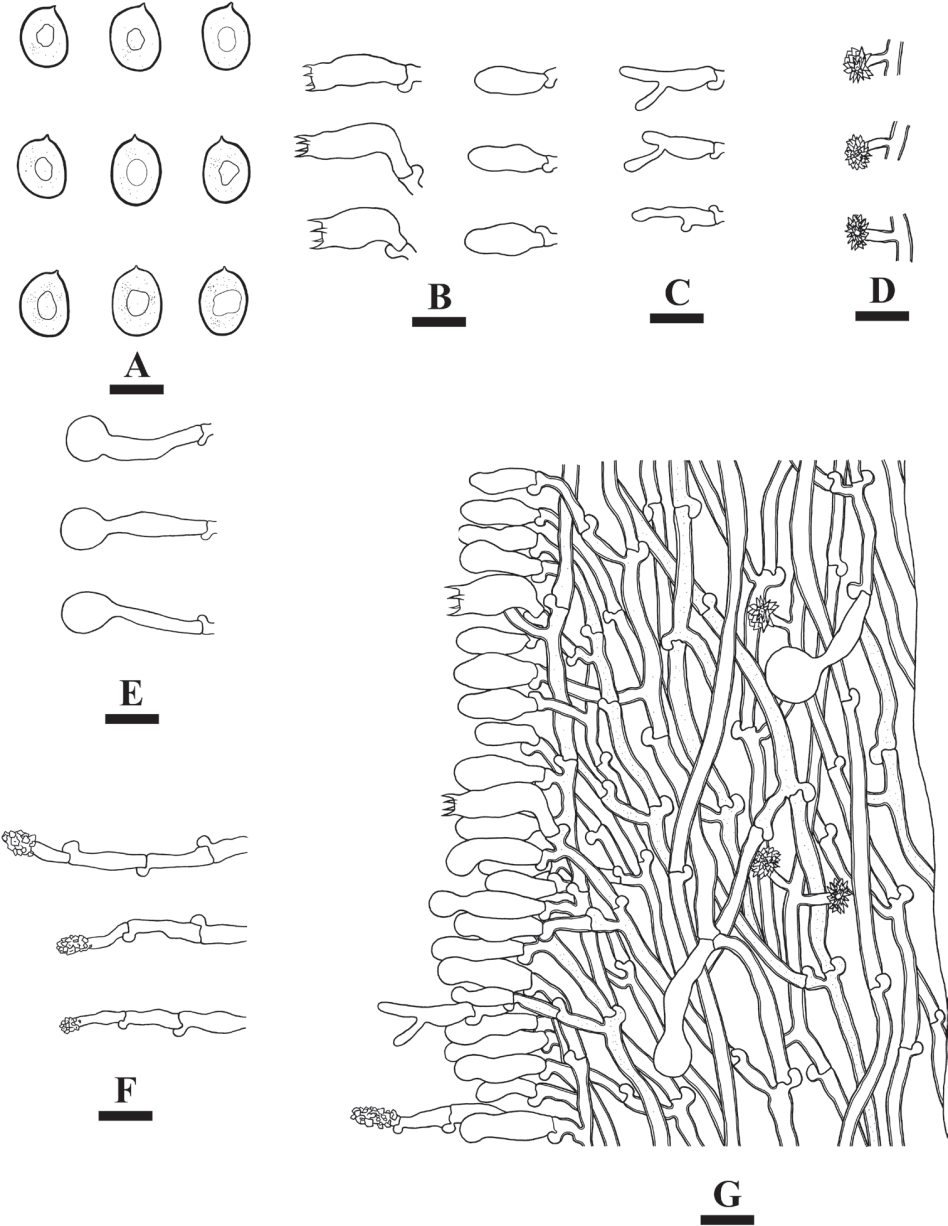
Microscopic structures of *Xylodonpuerensis* (holotype) **A** basidiospores **B** basidia and basidioles **C** paraphysoid cystidia **D** astrocystidium **E** capitate cystidia **F** septocystidium cystidia **G** a section of hymenium. Scale bars: 5 µm (**A**); 10 µm (**B–G**).

Basidiospores ellipsoid to broad ellipsoid, colorless, thin-walled, smooth, with oil drops, IKI–, CB–, (5.5–)6–7 × 4.5–5.5 µm, L = 6.41 µm, W = 5.01 µm, Q = 1.28 (n = 30/1).

##### Additional specimen examined

**(paratype).** China. Yunnan Province, Puer, Jingdong County, Taizhong Town, Ailaoshan Ecological Station, 24°29'41"N, 100°56'32"E, altitude 1930 m a.s.l., on angiosperm trunk, leg. C.L. Zhao, 24 August 2018, CLZhao 8639 (SWFC).

## ﻿Discussion

Many recently described wood-inhabiting fungal taxa have been reported in the subtropics and tropics, including in the genera *Lyomyces* and *Xylodon* (Xiong et al. 2009; Chen et al. 2017; Kan et al. 2017a, b; Riebesehl and Langer 2017; Viner et al. 2018; Chen and Zhao 2020; Luo et al. 2021a, b, c; Luo et al. 2022; Qu and Zhao 2022; Qu et al. 2022; Viner et al. 2022). Prior to this study, the following fourteen *Lyomyces* species were reported from China: *L.albus* (Sheng H. Wu) Riebesehl & Langer, *L.bambusinus*, *L.capitatocystidiatus* (H.X. Xiong, Y.C. Dai & Sheng H. Wu) Riebesehl & Langer, *L.cremeus* C.L. Zhao, *L.fissuratus*, *L.fumosus*, *L.leptocystidiatus* Xue W. Wang & L.W. Zhou, *L.macrosporus* C.L. Zhao & K.Y. Luo, *L.microfasciculatus* (Yurchenko & Sheng H. Wu) Riebesehl & Langer, *L.niveus*, *L.ochraceoalbus*, *L.sambuci*, *L.tenuissimus* (Yurchenko & Sheng H. Wu) Riebesehl & Langer and *L.wuliangshanensis* C.L. Zhao. (Xiong et al. 2009; Yurchenko et al. 2013; Riebesehl and Langer 2017; Chen and Zhao 2020; Luo et al. 2021b, c; Wang et al. 2021). The present study reports five new species in *Lyomyces* and *Xylodon*, based on a combination of morphological features and molecular evidence.

Phylogenetically, based on the multiple loci in *Hyphodontia* s.l., six genera, *Fasciodontia*, *Hastodontia*, *Hyphodontia*, *Lyomyces*, *Kneiffiella*, and *Xylodon*, were divided into four clades in the order Hymenochaetales (Wang et al. 2021). In the present study, based on the ITS+nLSU data (Fig. 1), *Lyomyces* and *Xylodon* were grouped with *Fasciodontia*, *Hastodontia*, *Hyphodontia* and *Kneiffielle*, in which five new species were grouped into the genera *Lyomyces* and *Xylodon*. Based on ITS topology (Figs 2, 3), *L.albopulverulentus* formed a monophyletic lineage, and was then grouped closely with *L.bambusinus*, *L.orientalis*, and *L.sambuci*. In addition, *L.yunnanensis* was found to be a sister to *L.niveus* with strong supports. The topology, based on ITS sequences, revealed that *X.daweishanensis* was retrieved as a sister to *X.hyphodontinus*. Moreover, *X.fissuratus* was grouped with the four taxa *X.montanus*, *X.subclavatus*, *X.wenshanensis*, and *X.xinpingensis*. *X.puerensis* was clustered with *X.flaviporus*, *X.ovisporus*, *X.subflaviporus*, *X.subtropicus*, and *X.taiwanianus*. However, morphologically, *L.bambusinus* can be delimited from *L.albopulverulentus* by its colliculose-to-tuberculate hymenial surface, its narrower basidia (16.5–35 × 3.5–7 µm), and its smaller and more broadly ellipsoid basidiospores (4.7–5.9 × 3.7–4.6 µm; Chen and Zhao 2020); Further, *L.orientalis* can be delimited from *L.albopulverulentus* by its smooth or slightly tuberculate hymenial surface, and by both its smaller basidia (13–20 × 3.5–4.5 µm) and basidiospores (5–6 × 4–4.5 µm; Yurchenko et al. 2017); *L.sambuci* can be delimited from *L.albopulverulentus* by its smooth-to-tuberculate hymenial surface and its smaller basidiospores (4.5–6 × 3.5–4 µm; Bernicchia and Gorjón 2010); *L.niveus* can be delimited from *L.yunnanensis* by a smaller basidia (9.5–15.0 × 3.5–5.5 µm) and broadly ellipsoid basidiospores (3.5–5 × 3–4 µm; Luo et al. 2021c). *Xylodonhyphodontinus* differs from *X.daweishanensis* by its larger basidiospores (4–5 × 4.5 µm; Hjortstam and Ryvarden 1980). *X.montanus* could be delimited from *X.fissuratus* by its smooth hymenial surface and moniliform cystidia (19.5–47.6 × 3.6–7.1 µm; Qu et al. 2022); *X.subclavatus* differs from *X.fissuratus* by its larger capitate cystidia (20–25 × 3–4 μm) and wider basidiospores (4–5.5 × 3.5–4; Yurchenko et al. 2013); *X.wenshanensis* can be delimited from *X.fissuratus* by its smaller capitate cystidia (6–11 × 3–6.5 µm; Luo et al. 2022); *X.xinpingensis* can be delimited from *X.fissuratus* by its reticulate hymenial surface and larger basidia (18.5–33 × 3–6.5 µm; Ma and Zhao 2021). *Xylodonflaviporus* differs from *X.puerensis* by its wider basidia (14.5–20 × 5–7 µm) and smaller basidiospores (4.5–5.5 × 3–3.5 µm; Ryvarden 1985); *X.ovisporus* differs from *X.puerensis* by its smaller basidiospores (3.5–4.3 × 2.8–3.3 µm; Riebesehl and Langer 2017); *X.subflaviporus* is distinguishable from *X.puerensis* by its narrower basidia (8–18 × 4–5 µm) and smaller basidiospores (3.9–4.8 × 2.7–3.5 µm; Chen et al. 2017); *X.subtropicus* differs from *X.puerensis* by its smaller basidiospores (5–5.8 × 3.5–4 µm; Chen et al. 2017); *X.taiwanianus* differs from *X.puerensis* by its smaller basidiospores (4.5–5.5 × 2.6–3 µm; Wu 2001).

Morphologically, *Lyomycesalbopulverulentus* resembles *L.bambusinus*, *L.cremeus*, *L.mascarensis* Riebesehl, Yurch. & Langer, *L.orientalis*, and *L.wuliangshanensis*, by sharing capitate cystidia and ellipsoid basidiospores. However, *L.bambusinus* differs from *L.albopulverulentus* by possessing a tapering cystidia (40–65 × 4–5.5 µm) and smaller basidiospores (4.7–5.9 × 3.7–4.6 µm; Chen and Zhao 2020); *L.cremeus* differs from *L.albopulverulentus* by its narrower capitate cystidia (20–40 × 3–5 µm), both smaller basidia (9–18.5 × 3–6 µm) and basidiospores (4.5–5.6 × 3.3–4.3 µm; Chen and Zhao 2020); *L.mascarensis* can be delimited from *L.albopulverulentus* by smaller capitate cystidia (17–38 × 3.5–6 µm), basidia (16–17.5 × 3.5–4.5 µm) and basidiospores (4.5–6 × 3.3–4 µm; Yurchenko et al. 2017); *L.orientalis* can be delimited from *L.albopulverulentus* due to its smaller capitate cystidia (17–38 × 3.5–6 µm), basidia (16–17.5 × 3.5–4.5 µm) and basidiospores (4.5–6 × 3.3–4 µm; Yurchenko et al. 2017); *L.wuliangshanensis* is different from *L.albopulverulentus* by smaller capitate cystidia (22–37 × 3–6 µm), basidia (12–20 × 3–4.3 µm) and basidiospores (3.5–5.3 × 2.8–4 µm; Chen and Zhao 2020).

Morphologically, *Lyomycesyunnanensis* resembles *L.bambusinus*, *L.cremeus*, *L.fumosus*, *L.fissuratus* and *L.wuliangshanensis* in both its capitate and tapering cystidia. However, *L.bambusinus* differs from *L.yunnanensis* by possessing a larger capitate cystidia (35–55 × 4–7 µm; Chen and Zhao 2020); *L.cremeus* differs from *L.yunnanensis* due to its smooth hymenial surface and smaller basidia (9–18.5 × 3–6 µm; Chen and Zhao 2020); *L.fissuratus* can be delimited from *L.yunnanensis* by its white-to-cream hymenial surface, and the presence of submoniliform cystidia (15.5–22 × 2.7–4 µm; Luo et al. 2021b); *L.fumosus* differs from *L.yunnanensis* due to its smooth hymenial surface, the presence of moniliform cystidia (8.5–22.7 × 2.5–3.7 µm), and its smaller basidia (11.5–17.5 × 3–5 µm; Luo et al. 2021b); *L.wuliangshanensis* is distinguishable from *L.yunnanensis* by its larger capitate cystidia (22–37 × 3–6 µm) and its smaller basidiospores (3.5–5.3 × 2.8–4 µm; Chen and Zhao 2020).

Morphologically, *Xylodondaweishanensis* is similar to *X.follis* Riebesehl et al., *X.grandineus* K.Y. Luo & C.L. Zhao, *X.laceratus* C.L. Zhao, *X.macrosporus*, *X.sinensis* C.L. Zhao & K.Y. Luo and *X.tropicus* C.L. Zhao due to its grandinioid, or odontioid, hymenial surface. However, *X.follis* differs from *X.daweishanensis* due to its cream hymenial surface, wider capitate cystidia (17–30 × 4.5–9 µm), and larger, globose to subglobose basidiospores (8–9.5 × 7–8.5 µm; Riebesehl et al. 2019); *X.grandineus* differs from *X.daweishanensis* by its subulate cystidia (11–19 × 3–5 µm; Luo et al. 2022); *X.laceratus* can be delimited from *X.daweishanensis* by its fusiform cystidia (20.3–26.8 × 5.3–6.4 µm) and its larger basidiospores (3.9–5.3 × 2.6–4.1 µm; Qu et al. 2022); *X.macrosporus* differs from *X.daweishanensis* by its cylindrical cystidia (44–79.5 × 3–6 µm), larger basidia (11.5–36 × 5–11 µm) and thick-walled basidiospores (8–10.5 × 7.5–9 µm; Luo et al. 2021a); *X.sinensis* differs from *X.daweishanensis* by its fusiform cystidia (10–21 × 3–6 µm), and its buff-to-brown hymenial surface (Luo et al. 2021a); *X.tropicus* can be delimited from *X.daweishanensis* by its subglobose, slightly thick-walled basidiospores (Qu et al. 2022).

*Xylodonfissuratus* resembles *X.attenuatus* Spirin & Viner, *X.borealis* (Kotir. & Saaren.) Hjortstam & Ryvarden, *X.bresinskyi* (Langer) Hjortstam & Ryvarden, *X.dimiticus* (Jia J. Chen & L.W. Zhou) Riebesehl & E. Langer, *X.grandineus* and *X.vesiculosus* Yurchenko et al. by it sharing similar ellipsoid basidiospores. However, *X.attenuatus* differs from *X.fissuratus* due to its odontoid hymenial surface, the presence of hyphoid cystidia (17.6–39 × 2.7–4.6 µm) and its larger capitate cystidia (14.2–27.2 × 3.3–4.5 µm; Viner et al. 2018); *X.borealis* differs from *X.fissuratus* by its slender hypha-like cystidia (40–70 × 3–5 µm), larger capitate cystidia (20–50 × 4–6 µm) and basidia (15–20 × 4–5 µm; Bernicchia and Gorjón 2010); *X.bresinskyi* differs from *X.fissuratus* by its poroid hymenial surface with rudimentary console shaping (Langer 2000); *X.dimiticus* is distinguishable from *X.fissuratus* by poroid hymenial surface with angular pores (2–4 per mm; Chen et al. 2016); *X.grandineus* differs from *X.fissuratus* due to its subulate cystidia (11–19 × 3–5 µm) and its smaller basidiospores (3–4.5 × 2–3 µm; Luo et al. 2022); *X.vesiculosus* can be delimited from *X.fissuratus* by its odontioid hymenial surface and larger basidiospores (5.3–6.3 × 3–4 µm; Riebesehl et al. 2019).

*Xylodonpuerensis* is similar to *X.bresinskyi*, *X.dimiticus*, *X.hallenbergii* (Sheng H. Wu) Hjortstam & Ryvarden, *X.poroideoefibulatus* (Sheng H. Wu) Hjortstam & Ryvarden, *X.reticulatus* (C.C. Chen & Sheng H. Wu) C.C. Chen & Sheng H. Wu, *X.subtropicus* and *X.syringae* (Langer) Hjortstam & Ryvarden by sharing a similar poroid hymenophore. However, *X.bresinskyi* can be delimited from *X.puerensis* by possessing smaller basidiospores (4.5–5.5 × 3–3.5 µm; Langer 2000); *X.dimiticus* differs from *X.puerensis* by possessing smaller basidia (9–13 × 4.5–6 µm) and basidiospores (3.8–4.6 × 2.8–3.5 µm; Chen et al. 2016); *X.hallenbergii* can be delimited from *X.fissuratus* by its both smaller capitate cystidia (15–23 × 4–5.3 µm) and basidiospores (4.2–5 × 4–4.3 µm; Wu 2001); *X.poroideoefibulatus* differs from *X.puerensis* by possessing smaller capitate cystidia (12–23 × 5.5–6.5 µm) and basidiospores (5–5.7 × 4–4.5 µm; Wu 2001); *X.reticulatus* can be delimited from *X.puerensis* by possessing smaller basidiospores (5–5.5 × 3.5–4 µm; Wu 1990); *X.syringae* differs from *X.puerensis* by its larger basidia (20–32 × 4–5 µm) and suballantoid basidiospores (8–9 × 3–3.5 µm; Hjortstam and Ryvarden 2009).

## Supplementary Material

XML Treatment for Lyomyces albopulverulentus

XML Treatment for Lyomyces yunnanensis

XML Treatment for Xylodon daweishanensis

XML Treatment for Xylodon fissuratus

XML Treatment for Xylodon puerensis
